# Connectomes: from a sparsity of networks to large-scale databases

**DOI:** 10.3389/fninf.2023.1170337

**Published:** 2023-06-12

**Authors:** Marcus Kaiser

**Affiliations:** ^1^NIHR Nottingham Biomedical Research Centre, School of Medicine, University of Nottingham, Nottingham, United Kingdom; ^2^Sir Peter Mansfield Imaging Centre, School of Medicine, University of Nottingham, Nottingham, United Kingdom; ^3^School of Medicine, Shanghai Jiao Tong University, Shanghai, China

**Keywords:** connectome, brain connectivity, databases, comparative connectomics, network science

## Abstract

The analysis of whole brain networks started in the 1980s when only a handful of connectomes were available. In these early days, information about the human connectome was absent and one could only dream about having information about connectivity in a single human subject. Thanks to non-invasive methods such as diffusion imaging, we now know about connectivity in many species and, for some species, in many individuals. To illustrate the rapid change in availability of connectome data, the UK Biobank is on track to record structural and functional connectivity in 100,000 human subjects. Moreover, connectome data from a range of species is now available: from *Caenorhabditis elegans* and the fruit fly to pigeons, rodents, cats, non-human primates, and humans. This review will give a brief overview of what structural connectivity data is now available, how connectomes are organized, and how their organization shows common features across species. Finally, I will outline some of the current challenges and potential future work in making use of connectome information.

## 1. Introduction

Crick and Jones (1993) noted that early data on neural network connectivity was limited to small circuits and few species. However, the field of connectomics has rapidly expanded since then. In 2005, a search in PubMed yielded only one new article per week on brain connectivity, but now there are over 50 articles on the topic. Brain connectivity, particularly functional connectivity, has replaced brain activity as the primary abstract keyword for the annual Human Brain Mapping conference. In this review, we will mainly focus on structural connectivity, although functional and structural connectivity are closely related.

Recently, there has been a wealth of connectome information on large cohorts of human subjects and patients with varying stages of brain development or disease progression (Kaiser, 2020). The Human Connectome Project in the USA has led to the availability of large datasets of brain networks in health and disease, while the UK Biobank Imaging project is collecting structural and functional brain connectivity data from 100,000 subjects. Additionally, longitudinal studies on brain development from before birth to early childhood and throughout the lifespan are being conducted.

Efforts are underway to enhance data quality, facilitate data sharing, and analyze brain network architecture. This review examines the available structural connectome data in different species, the establishment of databases, and the future challenges in the field.

## 2. Varying connectomes between species

The following sections will present species for which information about brain connectivity is available. Since the discovery of the meso-scale connectome of the round worm *Caenorhabditis elegans* (White et al., 1986), we have expanded our understanding to the much more complex brains of the fruit fly, pigeon, mouse, rat, ferret, cat, rhesus monkey, marmoset monkey, and human. With the growing number of species covered, comparative connectomics has become a possibility (van den Heuvel et al., 2016). All of these networks follow common wiring principles such as a modular organization, network hubs, and directed links (Kaiser, 2015). Additionally, studies have shown that, similar to *C. elegans*, macaque, and human, structural connectivity features non-optimal component placement which incurs higher energy costs for connection establishment and maintenance but enables a wider range of brain network dynamics (Kaiser and Hilgetag, 2006; Hayward et al., 2023). Moreover, a comparative study of 123 mammalian connectomes has shown that species with fewer interhemispheric connections exhibit better intra-hemispheric connectivity (Assaf et al., 2020).

Note that most of the networks discussed in this review are available for download here^1^ as part of the Open Connectome Project,^2^ or from databases mentioned for an individual species.

### 2.1. The worm (*Caenorhabditis elegans*)

**FIGURE 1:**
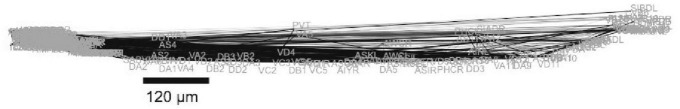
Lateral view of the neuronal network in *Caenorhabditis elegans*, with ganglia in the head region (on the left side) and the ventral cord visible at the bottom (after Kaiser and Hilgetag, 2006).

Belonging to the animal group Nemathelminths, the roundworms or nematodes have a constant number of neural cells for each species (eutelic). These creatures have an unsegmented body, so any movement involves the entire body. One unique aspect of their motor system is that muscle cells extend toward the axon to form a synaptic connection. *C. elegans*, a type of roundworm, has been the subject of genetic research for studying the connections between genes and behavior and development. In the hermaphrodite form of *C. elegans*, there are 302 neurons, while in the male form, there are 381 neurons. Moreover, scientists have discovered the complete cell lineage (Durbin, 1987), the connectivity of neurons (White et al., 1986), and their spatial positions (Choe et al., 2004) (refer to Figure 1). *C. elegans* is the only animal for which a complete connectome is available.

Emile Maupas first described *C. elegans* in 1900 (Blaxter, 2011). This worm is transparent during all stages of development, making it ideal for studying development. The embryos go through a stereotypical pattern of cell division from the zygote to the larva stage, and the cell lineage is mostly invariant (Sulston and Horvitz, 1977; Sulston et al., 1983). Therefore, we can observe the formation of the nervous and other systems over time. An adult hermaphrodite has 959 cells, excluding the germline, while a hatching larva has only 558 cells.

The nervous system of *C. elegans* is characterized by a longitudinal bundle of fibers in the ventral cord of the animal and several ganglia with a higher density of neural cell bodies (White et al., 1986; Hall and Altun, 2008). For instance, the diffuse pharynx “ganglion” envelops the pharynx musculature and follows its contours. The anterior and lateral ganglia surround portions of the pharynx muscles and neurons, while the small dorsal ganglion lies partially above the lateral ganglion. The ventral ganglion lies below the lateral ganglion, and in the tail, some of the small dorsorectal ganglion lies over the anterior portion of the lumbar ganglion (Cherniak, 1994).

### 2.2. Fruit fly (*Drosophila melanogaster*)

Insects belong to the arthropod animal group and have been around for over 400 million years. They have multiple units in their extremities and a variety of sensory organs to detect mechanical, olfactory, and visual stimuli. Transmitting information quickly from their heads to their legs presents a challenge due to their larger size compared to *C. elegans*. Unlike myelinated axons, invertebrates such as insects use giant axons with a larger diameter to increase conduction speed. Fruit flies like *Drosophila melanogaster* are commonly used in genetic studies due to their small size and short generation cycle.

The central brain of *Drosophila melanogaster* comprises approximately 135,000 neurons, significantly more than the *C. elegans* nervous system, which has only about 300 neurons, but considerably fewer than the mouse brain with over 100 million neurons or the macaque brain with over 1.3 billion neurons. Shih et al. (2015) established the FlyCircuit database, which contains data from 12,995 projection neurons based on confocal microscopy. These neurons are organized into 58 bundled neural tracts, which link 49 functional units. The 49 units are classified into five modules: olfactory, mechano-auditory, left visual, right visual, and pre-motor. This functional specialization is also observed in mammalian brains, such as the cat brain, which is composed of visual, auditory, somatosensory-motor, and fronto-limbic modules (Scannell et al., 1995).

Although functional modules in the brain allow for the separation of information processing, it is also crucial to integrate different types of information. Shih et al. (2015) discovered that certain nodes within these modules have significantly higher connection strengths than others, suggesting their role as integrators or broadcasters of information. These nodes coordinate the flow of information locally within modules and assist in connecting information from various modules on a global scale. This process of local and global integration is analogous to the functions of highly connected hubs, both provincial and connector types.

Shih et al. (2015) found that highly connected nodes, which were measured by the total strength of connections rather than the number of connected nodes (node degree), have stronger connections between each other than would be anticipated. This “rich-club” organization (van den Heuvel and Sporns, 2011), where strong links exist between highly connected nodes, facilitates synchronization and global information integration.

Similar to other species, from *C. elegans* to macaques, the direction of connections is also recorded in the fly dataset. However, these connections often exhibit asymmetry, where one direction might be considerably weaker or absent compared to the other, resulting in one-way streets of information flow. This asymmetry could potentially contribute to the formation of functional circuits with distinct feedforward and feedback loops, which might be influenced by differences in developmental time windows for synapse formation (Lim and Kaiser, 2015).

It is important to note that there are some differences between the organization of the arthropod *Drosophila*’s brain and mammalian brains, beyond the size of the brains. For example, interneurons and motor neurons’ somata are typically uni-polar in *Drosophila*, and motor fiber bundles are not myelinated. Additionally, *Drosophila* has sensory organs, such as compound eyes, ocelli, and antennae, which lack obvious counterparts in mammals. Furthermore, the current method used cannot resolve individual synapses, and higher-resolution methods are necessary to obtain information about synaptic weight, the location of synapses with excitatory versus inhibitory effects in the post-synaptic neuron, and the computation ability within the dendritic tree of a neuron.

### 2.3. Pigeon (*Columba livia*)

The avian class, Aves, originated over 150 million years ago and encompasses approximately 8,800 species. They possess unique adaptations, including modifications in their nervous system, which allow them to fly by reducing their body weight.

Song-learning birds exhibit a distinct pattern of cell birth and cell death during adulthood (Nottebohm, 2005). The pallial song control nucleus HVC experiences a reduction in neuron count between breeding seasons but gains almost 68,000 new neurons during breeding seasons (Larson et al., 2014). Similarly, food-storing birds exhibit seasonal changes in the size and neurogenesis of the hippocampus, which is associated with the retrieval of food cache locations (Sherry and Hoshooley, 2010).

**FIGURE 2:**
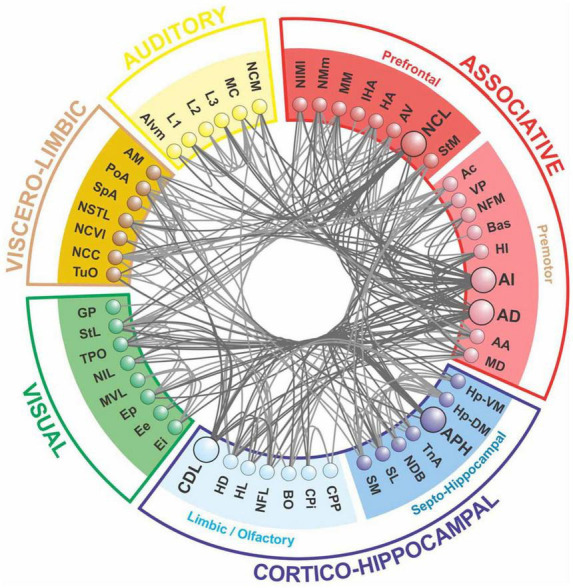
The forebrain connectome of pigeons is composed of five main modules, as revealed by network analysis. The associative and cortico-hippocampal modules can be further subdivided. Hub nodes are depicted in a darker shade, with connections to and from these nodes highlighted (after Shanahan et al., 2013).

The only available bird connectome is the fiber tract network between brain regions in the telencephalon of the pigeon (Shanahan et al., 2013), as shown in Figure 2. It displays typical network properties found in mammals, such as modularity, the presence of hub nodes, and structural motifs, facilitating both segregation and integration (Sporns and Kötter, 2004; Zamora-Lopez et al., 2010; Sporns, 2013).

The pigeon connectome shows a two-level modularity, with the top-level modules being functionally analogous to those of humans and primates. However, the pigeon’s top-level modules are more anatomically distributed than those of humans. The network also possesses a central connective core, which has higher betweenness centrality, node degree, and rich-club features. Hub nodes within this core are functionally similar to those in the primate brain (Shanahan et al., 2013). The pigeon connectome’s topological modules are comparable to those identified in human brain networks (Hagmann et al., 2008; Shanahan et al., 2013), including the prefrontal, premotor, and motor fields, the occipital visual module, and the viscero-limbic module (Güntürkün, 2005; Hagmann et al., 2008). Additionally, the cortico-hippocampal module includes areas of the hippocampal complex, as well as primary and associative sensory systems.

### 2.4. Mouse (*Mus musculus*)

About 65 million years ago, mammals underwent a rapid diversification resulting in over 4,000 species. For genetic and pharmacological studies, mice are a popular choice among rodents. With a weight of 0.4 g and containing 75 million neurons, the mouse brain is lissencephalic and lacks the folded cortical surface of other mammals.

The Allen Mouse Brain Connectivity Atlas project (Oh et al., 2014) and the Mouse Connectome Project reconstructed the connectivity of the C57BL/6 mouse brain. Initially, the mouse connectome (Zingg et al., 2014) reconstructed 240 intracortical connections forming a cortico-cortical connectivity map that allows for the comparison of connections from different cortical targets. Connectivity matrices were generated to provide an overview of all intracortical connections and subnetwork clusterings. The connectivity matrices and cortical map revealed that the entire cortex is organized into four somatic sensorimotor, two medial, and two lateral subnetworks that display unique topologies and can interact through select cortical areas. These data represent a valuable resource for investigating cortical networks and their corresponding functions.

Anterograde viral tract-tracing data provided by the Allen Institute for Brain Sciences (Oh et al., 2014) was used in a statistical approach (Ypma and Bullmore, 2016) to estimate that the connection density of the mouse intra-hemispheric cortical network is 73%, while the inter-hemispheric density is 59%. The weakest estimable connections, which may represent only one or a few axons, are about 6 orders of magnitude weaker than the strongest connections.

Similar to *C. elegans* (Kaufman et al., 2006), gene expression is a predictor of connectivity in mice (Wolf et al., 2011). Outgoing (incoming) connectivity can be successfully predicted for 73% (56%) of brain regions with an overall accuracy level of 0.79 (0.83). It is important to note that the study used rat brain connectivity to compare with mouse gene expression as the mouse connectome was not yet available.

The synaptome, the distribution of synapse types across different brain regions, has been mapped out (Zhu et al., 2018). Each brain region has a unique fingerprint of synapse types. Areas controlling higher cognitive function have the greatest synapse diversity, and mutations causing cognitive disorders reorganize synaptome maps. Additionally, new high-resolution optical methods may provide information about the network at the neuronal, micro-connectome level (Li et al., 2010), potentially linking synaptome and connectome information.

### 2.5. Rat (*Rattus norvegicus*)

The rat brain contains 200 million neurons and weighs 2 g. While it is larger than for mice and less commonly used for gene knockout research, new techniques may increase its utility in this area. Rats are often utilized for studying behavior related to memory and reward systems.

Early work by Burns and Young (2000) led to the mapping of 24 regions in the hippocampal formation and associated hippocampus through invasive tract tracing studies. Cluster analysis identified regions associated with “place” and “head-direction.” The *Brain Architecture Knowledge Management System* contains current information on the rodent macroconnectome, based on data from both rat and mouse (Bota et al., 2012). Unlike other connectomes, detailed information on sub-cortical structures, such as the basal ganglia (Swanson et al., 2016), intrinsic connectivity of the globus pallidus (Sadek et al., 2007), hippocampus (Ropireddy and Ascoli, 2011) and amygdala (Schmitt et al., 2012) as well as of cortical structures (Swanson et al., 2018), is available for rats.

There are several software environments available for visualizing and analyzing brain connectivity in rodents, including BrainMaps.org (Mikula et al., 2008), Brain Maps 4.0 (Swanson, 2018), the Neurome Project, and the Allen Institute Brain Atlas (Sunkin et al., 2013). NeuroVIISAS (Schmitt and Eipert, 2012) provides network analysis routines and the ability to interact with simulation tools like NEST (Gewaltig and Diesmann, 2007) to study network dynamics.

The rat connectome, which has been around for 14 years longer than the mouse connectome, has been extensively analyzed using these tools. According to recent studies (Swanson et al., 2018), at least 10,000 macroconnections exist between the 244 gray matter regions identified in the right and left cerebral hemispheres of the rat. Multiresolution consensus clustering (MRCC) revealed four subsystems at the top level of the hierarchical network analysis. In addition, the connectivity hub status of a region depends on the size and coverage of its anatomical neighborhood.

### 2.6. Ferret (*Mustela putorius furo*)

Ferrets, the domesticated version of European polecats, have brains that weigh 3.1 g and comprise 400 million neurons. These carnivores belong to one of the oldest recent groups, with males being considerably larger than females. Having a lower neural density than their cortical mass suggests, due to feeding on smaller prey and having less available energy, ferrets have fewer neurons in their brains (Jardim-Messeder et al., 2017).

Ferrets offer distinct advantages for developmental studies compared to other model systems. Due to slower brain development, many processes that occur before birth in other species, such as cortical folding, take place later in ferrets. This provides an opportunity to observe the effect of postnatal interventions, such as lesions, on ongoing brain development (Sukhinin et al., 2016). Additionally, ferrets share significant homologies with cats (Manger et al., 2010), which have a connectome available. Electrophysiological studies have also linked neural dynamics to behavior, making ferrets a valuable model system for the anatomical and functional study of early brain development.

Regarding the ferret connectome, researchers can access the Ferretome database through a web interface to search for tract tracing and cytoarchitecture data. The database includes data from 150 studies and contains 20 distinct injection sites with 200 labeled sites, as well as cytoarchitecture data for 12 regions, primarily in the visual and auditory cortex (Sukhinin et al., 2016).

### 2.7. Cat (*Felis silvestris catus*)

The brain of a domestic cat weighs 25–30 g, which is about 1% of its body mass, and is composed of approximately 1.2 billion neurons (Jardim-Messeder et al., 2017). Besides having a sophisticated visual system, cats are also susceptible to “human” conditions like cognitive decline and dementia.

**FIGURE 3:**
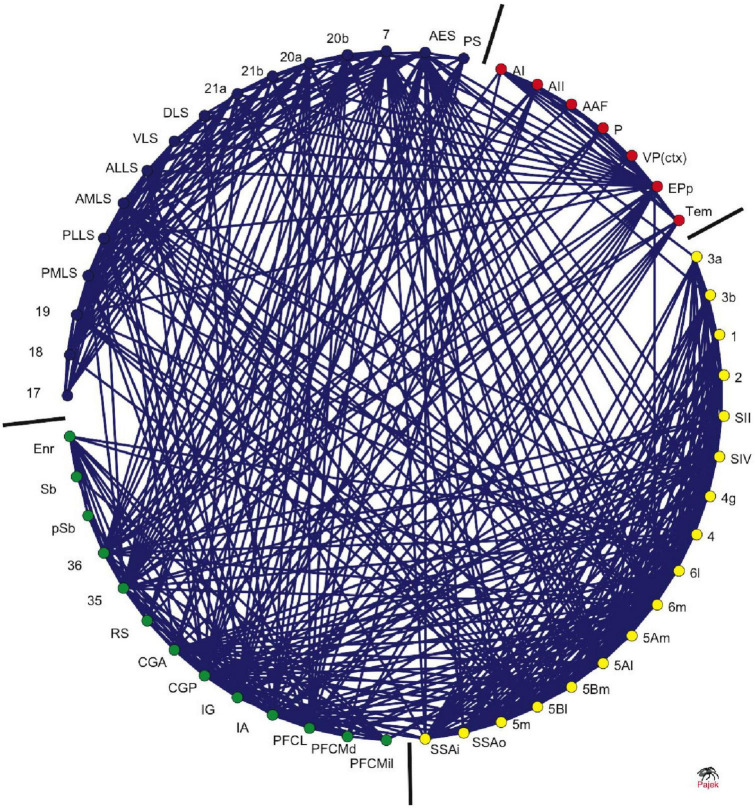
Clustered organization of cat corticocortical connectivity. The circular arrangement of cortical areas was optimized using an evolutionary algorithm to place highly interconnected areas closer to each other. The bars demarcate the borders between nodes in different clusters, which correspond to visual (blue), auditory (red), somatosensory-motor (yellow), and frontolimbic (green) cortices. The ordering of the nodes around the circle is consistent with their functional and anatomical similarities [adapted from Hilgetag and Kaiser (2004)].

The cat macro-connectome, which considers cytoarchitecture and physiology for cortical parcelation, is based on one hemisphere and includes 65 regions with 1,139 projections among them (Scannell et al., 1995, 1999). The strength of a connection is evaluated on an ordinal scale, with “1” indicating a weak connection, “2” indicating a medium-strength connection, and “3” indicating a strong connection. An investigation into topological modules (Hilgetag et al., 2000) identifies four modules linked to visual, auditory, fronto-limbic, and somatosensory-motor function (see Figure 3).

The cat connectome exhibits a rich-club organization with hubs located mostly at the boundaries between modules, according to de Reus and van den Heuvel (2013). Moreover, 86% of the connections between modules consist of rich-club connections that link rich-club nodes and feeder connections that link non-rich-club nodes to rich-club nodes.

Diffusion tensor imaging studies have also been conducted on structural connectivity. In new-born cats, the thalamo-cortical tract’s main body was smooth, and branching fibers were nearly straight, while the main body became more complex and branching fibers became curved in older cats, reflecting gyrification. Temporal lobe cortico-cortical tracts were smooth in new-borns, but they formed a sharper angle in later developmental stages. The cingulum bundle and superior longitudinal fasciculus became more visible over time. According to Takahashi et al. (2010), structural changes occurred in these tracts within the first month after birth, coinciding with the formation of gyri.

As with dogs, cats can exhibit cognitive decline, Cognitive Dysfunction Syndrome (CDS), and dementia, including beta-amyloid plaques and neurofibrillary tangles, making them potential animal models for Alzheimer’s disease. Therefore, it would be interesting to see future diffusion imaging studies conducted across the lifespan of cats.

### 2.8. Rhesus monkey (*Macaca mulatta*)

Rhesus monkeys, which belong to the macaque species, are omnivorous and old-world monkeys. They are indigenous to northern India, Myanmar, Southeast Asia, and eastern China. Their brain weighs approximately 87 g and contains 6.3 billion neurons (Herculano-Houzel et al., 2007). These monkeys are commonly used in studies of neural mechanisms of cognition and clinical applications such as polio vaccine development and deep brain stimulation. Macaque monkeys have been the primary focus of research, with some studies also conducted on marmosets, squirrel monkeys, and capuchin monkeys. However, great apes such as chimpanzees and lesser apes such as gibbons have not been studied in this context (Passingham, 2009).

The first comprehensive overview of macaque cortical connectivity, with a specific focus on the visual system, was published in 1991 (Felleman and Van Essen, 1991). Connections were classified on an ordinal scale of “1” for weak, “2” for intermediate, and “3” for strong connections, based on dozens of individual tract tracing studies. Additional studies were later incorporated into CoCoMac, the first online database of connectome data, which was developed in 1996 (Stephan et al., 2001; Kötter, 2004; Stephan, 2013).

Markov et al. (2013a,b, 2014) recently reported a systematic study of projections between 29 regions in the same hemisphere and to some regions in the contralateral hemisphere. Using retrograde tracer injections in 29 of the 91 areas of the macaque cerebral cortex revealed novel pathways absent in CoCoMac. The connectivity profile for each area conformed to a lognormal distribution, where a majority of projections are moderate or weak in strength. Importantly, this dataset highlights an unexpectedly high incidence of unidirectional links.

The macaque connectome’s visual system was analyzed using topological cluster analysis, revealing two distinct streams that correspond to the ventral and dorsal visual pathways for object recognition and object position/movement, respectively (Young, 1992). Further analysis identified two topological modules: the occipito-parietal and parietal modules, as well as the inferior-temporal and prefrontal modules, while the primary visual cortex, area V1, was separated from both (Hilgetag et al., 2000).

In addition, neuroimaging-based information on macaque connectivity is becoming increasingly available. The PRIME database, created by 22 non-human primate imaging sites, is gathering structural scans and diffusion tensor imaging data (Milham et al., 2018). The initiative also plans to expand its focus to other species, such as marmosets.

### 2.9. Marmoset monkey (*Callithrix jacchus*)

Marmosets, with their lissencephalic brain, twin births as a norm, and transgenic animal development, are more amenable to certain experimental procedures than other animals (Lin et al., 2019). Their brain is smaller (approximately 35 mm × 25 mm × 20 mm) and comparable in size to some rodents like squirrels, and their brain mass and number of neurons are only 10% of that of macaques (Herculano-Houzel et al., 2007).

**FIGURE 4:**
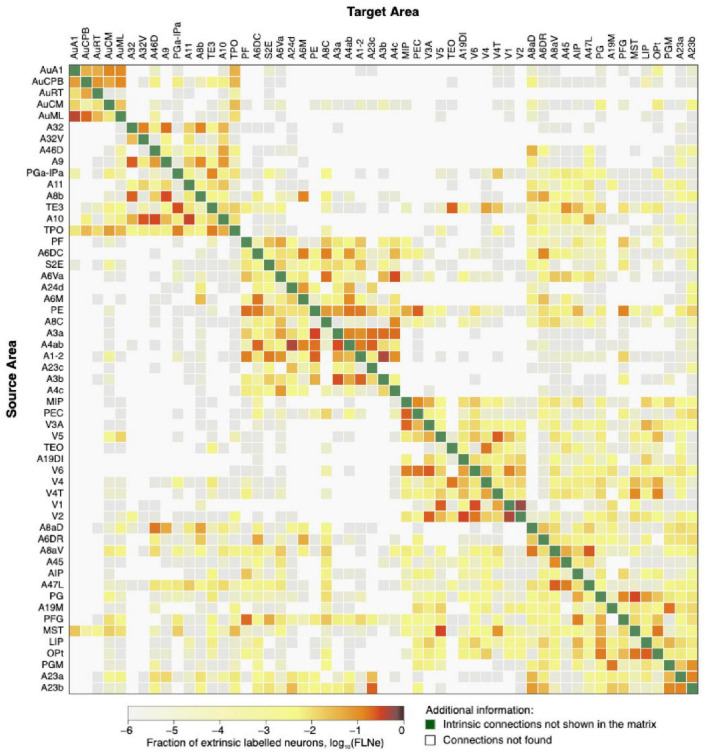
The marmoset structural connectivity is presented as a directed and weighted connectivity matrix that is based on the results of monosynaptic retrograde fluorescent tracer injections. The cortical areas are arranged in rows and columns, following hierarchical clustering. The injected areas are indicated as targets, while the areas from which the projections originate are listed as sources. This image was generated from the data available at http://analytics.marmosetbrain.org/, which is described in detail in the studies by Majka et al. (2016) and Lin et al. (2019).

The common marmoset (*Callithrix jacchus*), which is native to South America, has a database of about 50 cortical areas with tracer injection studies available online (Majka et al., 2016). Recent studies suggest that the visual, auditory, and motor systems of marmosets are highly similar to those of macaques (Bakola et al., 2015). Figure 4 presents a connectivity matrix displaying the fiber tract information in both directions using anterograde and retrograde tracers between all regions.

### 2.10. Human (*Homo sapiens*)

At birth, the human brain weighs approximately 350 g, which increases to around 1,000 g by 1 year of age, and to 1,300 g during puberty. The adult human brain weighs around 1,500 g and contains 86 billion neurons. Despite ample information available for other species, data on human neuroanatomy has been limited (Crick and Jones, 1993), leading to a proposal in 2005 to determine the human connectome in terms of connectivity between brain regions and neurons (Sporns et al., 2005).

**FIGURE 5:**
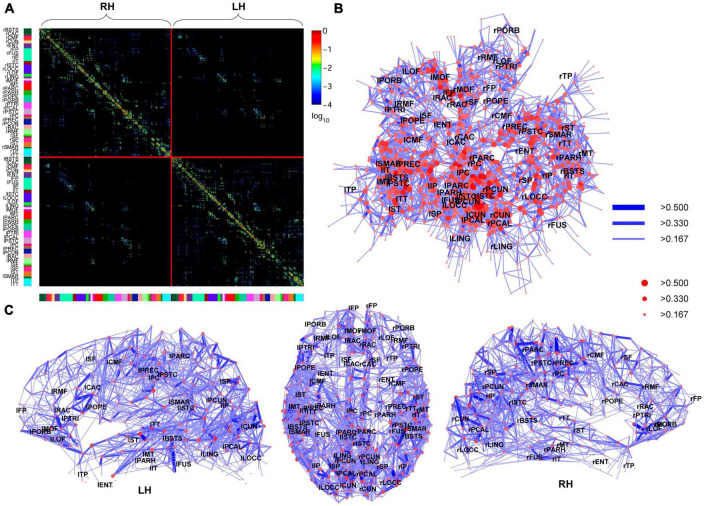
High-resolution human connectome matrix, network layout and connectivity backbone in one subject. **(A)** Matrix of fiber densities between all pairs of *n* = 998 regions of interest (ROIs) plotted by cerebral hemispheres. **(B)** The connectivity backbone is displayed using a Kamada-Kawai force-spring layout, with labels indicating anatomical subregions at their respective centers of mass. All connections are symmetric and displayed with a logarithmic color map. Nodes (ROIs) are coded based on strength and edges based on connection weight. **(C)** Dorsal and lateral views of the connectivity backbone with the same node and edge coding as in the previous subplot. From Hagmann et al. (2008).

In 2008, using non-invasive neuroimaging techniques, Hagmann et al. (2008) constructed the first macro-connectome. They used diffusion spectrum imaging to non-invasively map structural connectivity within and across cortical hemispheres in individual human participants, revealing a structural core in the posterior medial and parietal cerebral cortex. This core is characterized by nodes with high degree, high connection strengths, and high betweenness centrality. It includes brain regions that form the posterior components of the human default mode network and acts as a connector hub linking all major structural modules. The researchers found a high correspondence between structural connectivity and resting-state functional connectivity measured in the same participants, both within and outside of the core regions. Figure 5 illustrates this connectivity.

The initial results on human brain connectivity (Hagmann et al., 2008) were obtained from a small sample of five subjects. As each individual’s brain connectivity is as unique as a fingerprint, larger datasets are necessary to fully comprehend the relationship between network structure and function. To address this, the Human Connectome Project (HCP) was launched (Van Essen et al., 2012), which utilized a standardized approach to generate structural connectivity data (Glasser et al., 2013). The HCP collected data from 1,200 healthy participants, including adults, young adult twins, and non-twin siblings, to establish a baseline for human brain network organization. Following the success of the HCP, other similar projects have emerged, such as the Developing Human Connectome Project (Fitzgibbon et al., 2016) and various disease-related initiatives.

The UK Biobank Imaging project is an even larger-scale initiative (Miller et al., 2016). It has assembled data from 500,000 UK residents, including genetic information, blood samples, socio-economic data, healthcare records, and cognitive assessments. Among these subjects, 100,000 are undergoing MRI scans, including both structural (diffusion imaging) and functional (rs-fMRI) connectivity scans (Alfaro-Almagro et al., 2018). This dataset will include longitudinal data on dementia patients, allowing for the observation of disease progression and the development of early biomarkers, as many of these patients were symptom-free at the time of their initial UK Biobank scan.

## 3. Conclusion

Over the last 15 years, major databases of brain connectivity have been established. For many mammalian and invertebrate species, at least a connectome of one individual is available. However, there are efforts to measure brain connectivity in populations. For humans, large databases for healthy subjects and for individuals suffering from a range of brain and mental health conditions are being established. While such datasets were traditionally limited to Western populations (Ricard et al., 2023), there is great progress in including a wider range of developing and industrialized countries, e.g., large databases have been established in China (Xu et al., 2020; Ge et al., 2023). Moreover, data sets across the life span are becoming available (Zuo et al., 2017; Liu et al., 2021).

Apart from the information discussed in this article, there exist some other species for which partial knowledge about the nervous system is available, typically pertaining to the fibers connecting the brain to the sensory or motor systems. Some examples of such species are leeches, adult and larval tadpoles, among others, as documented by Borisyuk et al. (2008) and Ryan et al. (2016), respectively. Furthermore, there is more detailed connectome information available for specific brain subsystems, like the retina in mice, as demonstrated by Helmstaedter et al. (2013).

Today’s research is not any more restricted by the sparsity of available connectome information but concerns the ability to compare connectomes between studies and between species. Even within species, results are not always reproducible or generalizable, for example, due to suboptimal measurement reliability within individual samples (Zuo et al., 2019). Moreover, reporting the connectome of a species based on few sample brains might not give an accurate representation of brain organization and function (Marek et al., 2022).

As shown above, the definition of a connection can also vary depending on the method that was used to establish the connectome. At the same time, for reconstructing connectomes from diffusion imaging, networks can vary depending on the parcelation, imaging, and reconstruction parameters that were being used.

The generic organization of biological neural networks is remarkably similar across species showing hierarchical modularity, small-world features, and a rich-club organization. However, we increasingly notice how network differences between individuals are related to cognition and intelligence. It will be a challenge for the coming years to better understand how the unique fingerprint of the human connectome is linked to the behavior of the network. For this, while structural connectomes across species are becoming available, there is still a sparsity of functional connectome information across the animal kingdom. After all, there remains work to do, to better understand the relationship between brain structure and function.

## Author contributions

MK wrote the manuscript.

## Footnotes

1
https://connectomelab.github.io/changingconnectomes/chapter4/

2
https://neurodata.io/project/ocp/
